# Vitreous Hemorrhage following High-Altitude Retinopathy

**DOI:** 10.1155/2021/7076190

**Published:** 2021-08-10

**Authors:** Arjun Shrestha, Rinkal Suwal, Biju Shrestha

**Affiliations:** ^1^BP Eye Foundation, Hospital for Children, Eye, ENT & Rehabilitation Services, Bhaktapur, Nepal; ^2^Department of Physiology, Kathmandu Medical College, Kathmandu, Nepal

## Abstract

**Purpose:**

To report a case of high-altitude retinopathy with vitreous hemorrhage.

**Methods:**

An apparently healthy 29-year-old boy presented with a history of floater and slight diminution of vision in the left eye after climbing the mountain 4760 meters high.

**Results:**

The visual acuity at presentation was 20/20 in the right eye and 20/30 in the left eye. Anterior segment findings of both eyes were unremarkable. Fundus evaluation revealed bilaterally dilated major retinal veins. The right eye revealed clear, quiet vitreous, healthy macula, and pink and smooth optic disc. There was fresh vitreous hemorrhage confined just one disc diameter away at the superior and inferior part of the optic nerve in the left eye. The macula and optic nerve head of this eye grossly looked normal. Complete blood count, haemoglobin, ESR, CRP, sugar, renal function test, lipid panel, and serology for HIV, HCV, VDRL, and HBsAg were normal. The Mantoux test and chest X-ray also revealed normal findings. Physician consultation did not reveal other abnormalities. On the 3^rd^ week of follow-up, his vision was 20/20 in both eyes. Fundus examination revealed clear vitreous in both eyes though some venous dilation and tortuosities were still evident in the left eye. A macular OCT scan showed almost normal-appearing fundus.

**Conclusions:**

Vitreous hemorrhage following climbing high altitude can be one of the manifestations of high-altitude retinopathy.

## 1. Introduction

High-altitude retinopathy (HAR) is a manifestation of high-altitude sickness, with other presentations like high-altitude pulmonary edema, high-altitude cerebral edema, and acute mountain sickness. HAR constitutes numerous retinal vascular decompensations related to hypoxia, with developing sudden onset symptoms like headache, nausea, anorexia, insomnia, and fatigue. The acute ocular manifestations are mainly retinal hemorrhages, peripapillary hyperemia, and dilated vessels, which mostly occur in individuals with mountain climbers who are exposed to high altitude above 3,000 meters (m) [1].

Intra- and preretinal hemorrhages are generally seen at the peripheral retina. Macular and vitreous hemorrhage, papilledema, and cotton wool spots can also occur in severe cases. According to severity, Weidman and Tabin [2] have classified HAR in four different grades: grade one—dilated retinal veins with hemorrhages within one disc area, grade two—moderate dilated retinal veins with hemorrhages in 2 disc areas, grade three—advanced dilated retinal veins with hemorrhages in 3 disc areas and paramacular and vitreous hemorrhage, and grade four—engorged retinal veins with hemorrhages greater than three-disc areas, macular and vitreous hemorrhage, or papilledema.

At high altitudes, due to inadequate oxygen levels, the body's metabolic state demands a sequence of physiological changes, which consequently increases cardiac output and then pulmonary ventilation. HAR was first described by Singh et al. [3] in 1969, where they outlined the ocular deformities in 24 soldiers, accounted with high-altitude illness. They reported three cases with vitreous hemorrhage and four cases with papilledema. The etiopathogenesis of HAR is unknown. However, it is suggested that, like in other oxygen-dependent retinal diseases (e.g., von Hippel-Lindau disease, retinopathy of prematurity, proliferative diabetic retinopathy, and glaucoma), factors like heterodimeric transcription and hypoxia-inducible factor could be the mediator for the changes in HAR [4]. Here, we have reported a case of bilateral HAR in a mountain climber.

## 2. Case Report

We report a case of bilateral-altitude retinopathy with vitreous hemorrhage in the left eye in a 29-year-old boy. He presented to us with a history of floaters in the left eye after five days of climbing the Himalaya of 4760 meters in Nepal. He also complained of slight diminution of vision in the same eye. He disclosed that climbing mountain was his hobby and he was not using supplemental oxygen either. He denied a history of similar complaints in the past and did not give systemic symptoms like headache, giddiness, vomiting, or any discomfort.

On examination, the best-corrected visual acuity (BCVA) was 20/20 in the right eye and 20/30 in the left eye. Intraocular pressure was 12 mmHg in both eyes. Slit-lamp biomicroscopic examination of the anterior segment of both eyes revealed the normal finding. Dilated fundus examination revealed bilaterally dilated major retinal veins, and the retinal vein and artery ratio showed 3 : 1 in both eyes. The right eye revealed clear, quiet vitreous, healthy macula, and pink and smooth optic disc. There was fresh vitreous hemorrhage confined just one disc diameter away at the superior and inferior part of the optic nerve in the left eye. The macula and optic nerve head of this eye grossly looked normal. The peripheral retinal finding also revealed no tears or other abnormalities.

**Figure 1:**
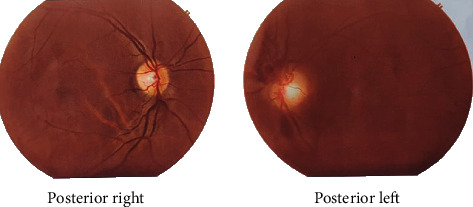
Fundus color photograph.

Color fundus photographs were taken and are shown in Figure 1. Macular optical coherence tomography (OCT) was not taken in this visit due to vitreous hemorrhage. Full blood count, haemoglobin, sugar, creatinine, urea, lipids, CRP, and serology for HIV, HCV, VDRL, and HBsAg were normal. The tuberculin test and chest X-ray also appeared normal. Consultation of an internal medicine specialist did not give any abnormalities.

The patient was counselled for a wait and watch schedule and asked to be followed up in 3 weeks or as soon as possible if flashes, curtain-like visual block, or poor vision was noticed.

**Figure 2:**
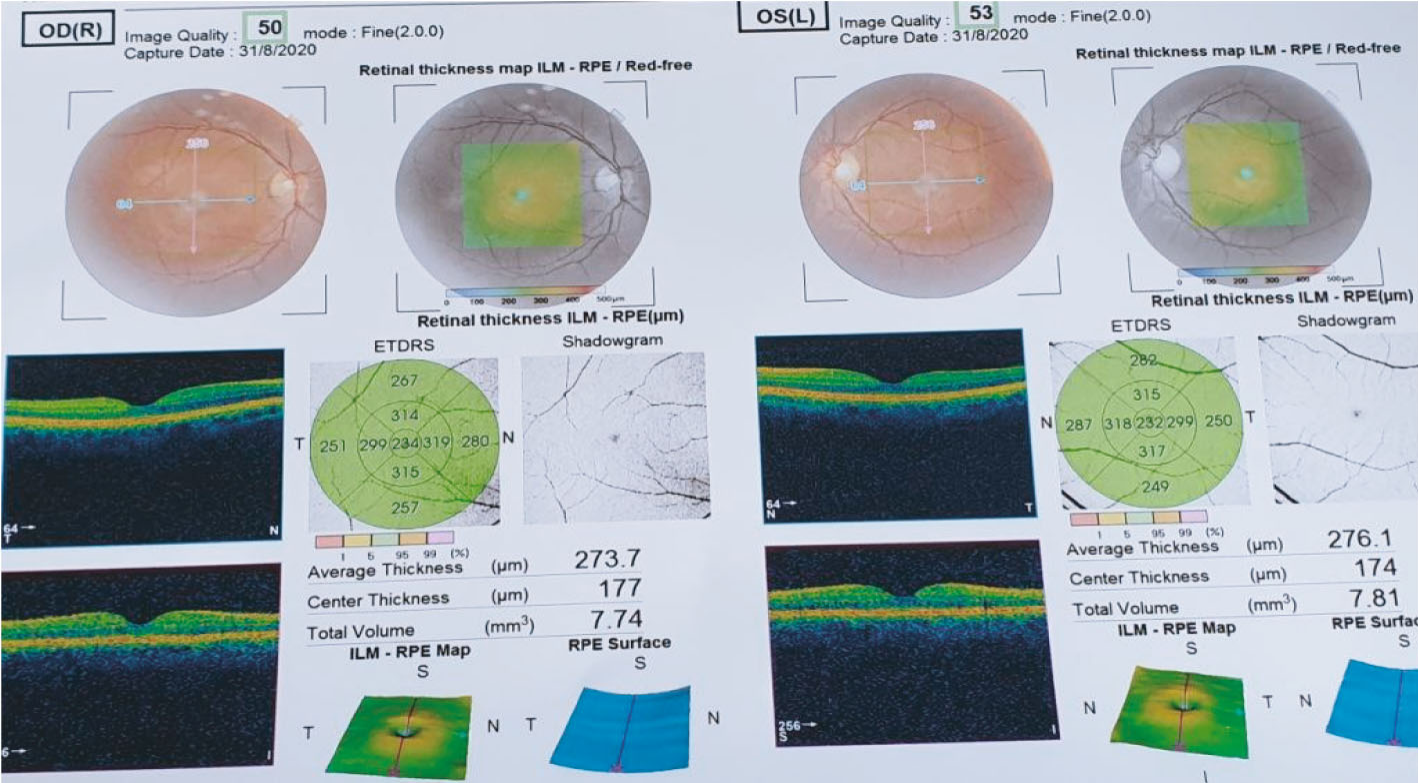
Macular OCT and color photograph showing normal findings.

On the 3^rd^ week, his BCVA was 20/20 in both eyes. Vitreous was clear in both eyes; however, retinal venous tortuosities and dilation were still present in the left eye. The macular OCT scan showed normal-appearing fundus. The retinal vein and artery ratio changed to 3 : 2. Color photos and OCT are shown in Figure 2.

## 3. Discussion

HAR represents numerous retinal vascular deformities associated with hypoxia. HAR is familiar findings among travelers at high altitudes, who exceed higher than 5000 m and are prone to developing severe high-altitude sickness like pulmonary edema, which could be life-threatening. Symptoms of individuals with HAR are mainly headache, insomnia, lassitude, and gastrointestinal upset. These symptoms generally begin from 8 to 24 hours and clear over four to eight days after exposure to high altitude [5].

Patients with HAR are generally presented with sudden diminution of vision after exposure to high altitude and then regress after travelling to a lower altitude. Nonetheless, HAR can be misdiagnosed. So, other causes should be ruled out before establishing a diagnosis of HAR because of similar presentations in other ocular pathology.

The retina has a higher metabolic activity than other human tissues. Furthermore, there is a vascular regulatory mechanism and close anatomical correlation on supplying vascular blood to both the brain and retina. Retinal changes in HAR are associated with possible ocular dysregulation, described in mountaineers with the increase in all hemodynamic parameters. However, in hypoxic conditions like in high altitudes, autoregulation of choroidal and retinal flow was stable in oxygen delivery. In addition, an increase in the level of hematocrit, which leads to an increase in blood viscosity, could also be one of the factors for causing HAR [6].

Available incidence has suggested that retinal hemorrhages are due to arteriole side leakage of the vascular bed or capillary rupture. Due to an increase in the volume and rate of blood flow, these capillaries might become more vulnerable. Increased intraocular pressure could also be a common cause of HAR for individuals after ascent to high altitude. Changes and deformities in retinal hemodynamics and hypoxia create several adjustments in circulatory and respiratory physiology, leading to increased cerebral blood flow and increased cerebral venous pressure [7]. These effects then decrease the cerebrospinal absorption, and then, hypoxia induces capillary permeability increasing cerebrospinal fluid resulting in papilledema. Hemorrhagic retinopathy is set because of an increase in retinal venous pressure. Yet the definite pathological changes are not still explained and remain an area for requiring further research.

HAR can be both mild and transient. Still, it could lead to permanent damage. A study done by Ho et al. [5] found that the BCVA was nearly restored in individuals with HAR to the baseline after three weeks. Yet there was the presence of vascular tortuosity and engorgement of the blood vessels in the retina. They also found defects in the retinal nerve fiber layer and optic nerve while examining through scanning laser polarimeter (GDx). Retinal vein occlusions have also been delineated in HAR [8]. In 1989, Butler et al. had done a prospective study on all fourteen individuals after six weeks of climbing Mount Everest. They reported intraretinal hemorrhages in five eyes in four climbers, and central retinal vein occlusion was found in one of these climbers. They suggested that increased intraocular pressure and usage of nonsteroidal anti-inflammatory drugs could be the significant risk factors for the occurrence of HAR [2]. Frayser et al. [9] also delineated retinal hemorrhages in nine out of twenty-five climbers ascending 5,335 meters on Mt. Logan. However, there was no relation between retinal hemorrhage and speed of ascent. In another study, retinal hemorrhages accounted for 36% of 39 individuals climbing above 4,329 m on Mt. McKinley [10]. Here, they found a positive correlation between the occurrence of retinal hemorrhage and the severity of headache. They also discovered that individuals with a history of migraine, increased physical exertion, and rapid ascent are more prone to developing retinal hemorrhages.

## 4. Conclusion

Individuals having ascents to high altitudes have a high risk of developing HAR. Therefore, a detailed ocular examination with proper history is needed for all of them.

## Data Availability

Clinical notes and fundus photos are available on reasonable request.

## References

[1] Seth R. K., Adelman R. A. (2010). High-altitude retinopathy and optical coherence tomography findings. *Seminars in Ophthalmology*.

[2] Wiedman M., Tabin G. C. (1999). High-altitude retinopathy and altitude illness. *Ophthalmology*.

[3] Singh I., Khanna P., Srivastava M., Lal M., Roy S. B., Subramanyam C. (1969). Acute mountain sickness. *New England Journal of Medicine*.

[4] Arjamaa O., Nikinmaa M. (2006). Oxygen-dependent diseases in the retina: role of hypoxia-inducible factors. *Experimental eye research*.

[5] Ho T.-Y., Kao W.-F., Lee S.-M., Lin P.-K., Chen J.-J., Liu J.-H. (2011). High-altitude retinopathy after climbing Mount Aconcagua in a group of experienced climbers. *Retina*.

[6] Lang G. E., Kuba G. B. (1997). High-altitude retinopathy. *American journal of ophthalmology*.

[7] Pugh L. G. C. E. (1962). Physiological and medical aspects of the Himalayan Scientific and Mountaineering Expedition. *British medical journal*.

[8] Arora R., Jha K., Sathian B. (2011). Retinal changes in various altitude illnesses. *Singapore Medical Journal*.

[9] Frayser R., Houston C. S., Bryan A. C., Rennie I. D., Gray G. (1970). Retinal hemorrhage at high altitude. *New England Journal of Medicine*.

[10] Schumacher G. A., Petajan J. H. (1975). High altitude stress and retinal Hemorrhage. *Archives of Environmental Health: An International Journal*.

